# Hand metastasis in a patient with cervical cancer: A case report

**DOI:** 10.1097/MD.0000000000020897

**Published:** 2020-07-02

**Authors:** Lenny Gallardo-Alvarado, Alma Astorga Ramos, Delia Perez-Montiel, Rebeca Ramirez-Morales, Erick Diaz, David Cantu-de Leon

**Affiliations:** a Department of Clinical Research, National Cancer Institute; b Medical Oncology Department, Mexican Institute of Social Security, Coahuila; c Department of Pathology, National Cancer Institute; d Department of Gynecology, National Cancer Institute, Mexico City, Mexico.

**Keywords:** acrometastasis, cervical cancer, rare metastasis

## Abstract

**Introduction:**

Acrometastasis is infrequent and generally indicates a wider spread of metastasis with poor prognosis. The diagnosis is challenging, as it might mimic an infectious, inflammatory, or metabolic disease. Acrometastasis are most commonly found in patients with lung, gastrointestinal, kidney, and breast cancer. Only 3 cases of cervical cancer associated with hand metastasis have been reported in the literature.

**Patient concerns:**

Herein, we report a 58-year-old patient with locally advanced cervical cancer and recurrence in the right thumb as presentation of widespread disseminated disease. She initially presented with adenocarcinoma of the uterine cervix and was treated with concurrent chemoradiation followed by high-dose rate brachytherapy. Six months later, she developed an insidious onset of pain and swelling in the right thumb, erythema, and edema, mimicking cellulitis.

**Diagnosis:**

A biopsy of the soft tissues of the thumb was performed, and the histopathology indicated metastasis of adenocarcinoma to the bone and soft tissues.

**Interventions and outcomes:**

The patient rejected further treatment and died of progressive disease 4 months after the diagnosis of the recurrence.

**Conclusion:**

Metastases in unusual sites are a diagnostic challenge, and there is no standardized treatment. Timely diagnosis and treatment can improve the prognosis of these patients and might preserve their quality of life.

## 1 Introduction

Metastases to the hand account for about 0.1% of all skeletal metastases. They are most commonly found in patients with lung, gastrointestinal, kidney, and breast cancer.^[1–7]^ Acrometastasis to the hand has rarely been reported in cervical carcinoma. A literature search revealed only 3 cases of hand metastasis of cervical carcinoma in 198 patients with hand metastasis, and only 4 out of 211 patients in an analysis of the hand and wrist metastasis by Afshar et al in 2014 had primary uterine cancer.^[1,2]^

Herein, we report a case of acrometastasis of cervical carcinoma as an early presentation of widespread disseminated disease.

## 2 Case report

**Figure 1:**
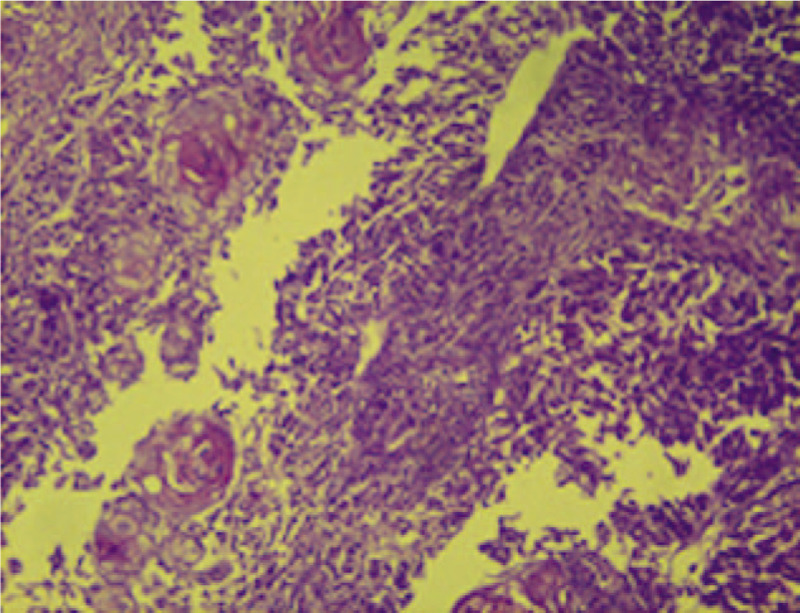
Cervical biopsy. Hematoxylin and eosin staining corresponding to a malignant neoplasm with a papillary growth pattern. Fibrovascular stalks show anaplastic epithelial lining surrounding groups of atypical squamous cells arranged cohesively, infiltrative cells, and vesicles with apparent nucleoli.

**Figure 2:**
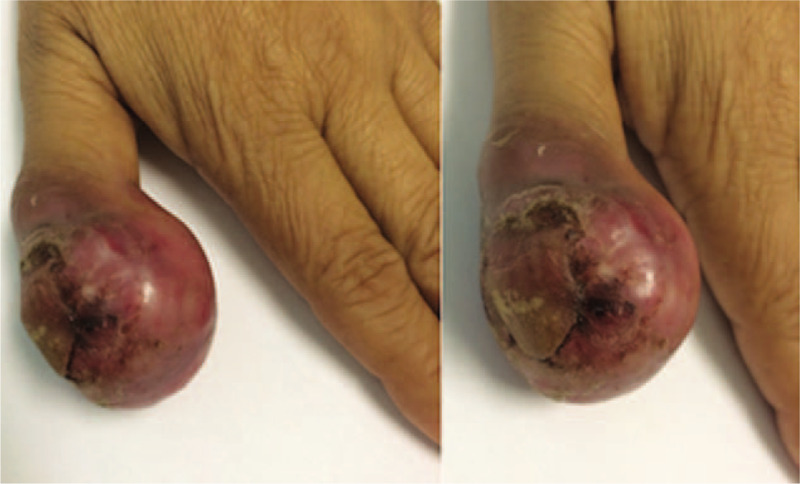
Images of cervical cancer thumb metastases. Dorsal and lateral view of the left thumb showing diffuse erythema and necrosis.

**Figure 3:**
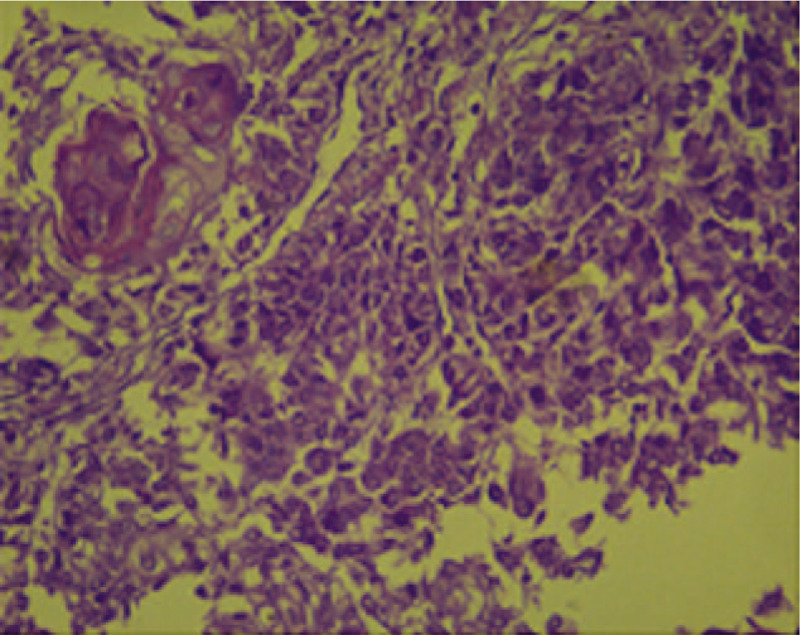
Thumb metastases. Hematoxylin and eosin stain of the thumb after amputation showing cells with a papillary growth pattern, anaplastic epithelial cells surrounding squamous cell groups, hyperchromatic cells characteristic of undifferentiated cells, pleomorphic cells with large cytoplasm, and vesicular nuclei with apparent nucleoli.

A 58-year-old woman presented with locally advanced cervical cancer (clinical stage IIB). The histologic type was moderately differentiated invasive adenocarcinoma (Fig. 1), and she was treated with concurrent chemoradiation (cisplatin, gemcitabine, and 45 Gy external beam radiotherapy) followed by high-dose rate brachytherapy. Six months after finishing treatment, she developed an insidious onset of pain and swelling in the right thumb, erythema, and edema, mimicking cellulitis. (Fig. 2) After excluding the possibility of an infectious or traumatic disease, a biopsy of the soft tissues of the thumb was performed. The histopathology indicated metastasis of adenocarcinoma to the bone and soft tissues. (Fig. 3)

The patient reported no other symptoms, and a work-up for distant disease was performed. Evidence of disseminated disease was confirmed; however, the patient rejected further treatment, and only palliative support was administered. Finally, she died of progressive disease 4 months after the diagnosis of recurrence.

Written informed consent was obtained from the patient for publication of the present report.

## 3 Discussion

The diagnosis of metastatic disease in this patient was prompt, by pathology analysis form the biopsy taken at the same time that other non-neoplastic pathologies were ruled out, our limitation was that we only give to our patients palliative and symptomatic treatment due to the patient's wishes.

Cervical carcinoma is the fourth most common malignancy in women worldwide. Moreover, clinical stage IV disease accounts for nearly 5% of new diagnoses of cervical cancer, and metastatic disease develops in 15% to 61% of women, usually within 2 years of completing primary treatment.^[8,9]^ The most common sites of relapse are locoregional structures such as the vaginal vault, endometrium, parametria, ovaries, fallopian tubes, and pelvic lymph nodes. Extrapelvic metastases frequently involve the paraaortic lymph nodes, lung, liver, and bones.^[1–3,5,10]^

Osseous metastases from cervical cancer have been reported in axial and peripheral bones and in all clinical stages. The contributing factors for bone metastasis are histology (it is more frequent in adenocarcinoma) and clinical stage (it is more common in advanced stages [II-IV]). The most common sites of skeletal metastasis are the lumbar and thoracic spine (more than 50%), followed by the pelvic bones (13%–20%). The principal mechanism of spreading is hematogenous.^[11–13]^

Hand metastases are rare, and although the mechanism is not well defined, the absence of bone marrow in the extremities may make them a hostile location for seeding of malignant cells. An increase in vascularity and trauma has been suggested as a possible mechanism.^[14]^ In most patients, the tumor initially metastasizes to the bone and spreads subsequently to the adjacent soft tissue. Acrometastasis often represents disseminated end-stage disease, and the patients consequently have a short life expectancy; the mean survival at the time of diagnosis is 7 months (range 3 to 18 months).^[3,14–18]^ The average age at presentation is 61 ± 13 years, with a male:female ratio of 2:1. The distal phalanx is involved most frequently, and the thumb is the most common site of presentation.^[1–3,8]^ The primary differential diagnoses are soft tissue infection, osteomyelitis, tenosynovitis, gout, complex regional pain syndrome, arthritis, and other inflammatory diseases.^[2]^

Acrometastasis to the hand has rarely been reported in cervical carcinoma. The primary tumor sites of acrometastasis are the lung (40%), gastrointestinal tract (25%), kidney (11%–13%), and breast (11%–50%).^[1–3,5,6,18]^ One report from 2004 showed only 3 cases related to cervical cancer (2%), and Afshar et al analyzed 221 cases of hand and wrist metastasis published over 27 years, of which uterine tumors accounted for only 2% (the study did not specify whether they were neoplasms of the endometrium or the cervix).^[1,2,5,6]^

Due to the rarity of acrometastasis, no standard treatment protocols exist. Most patients receive palliative treatment, because acrometastasis generally indicates a terminal disease with limited life expectancy. Early recognition is essential to ensure adequate treatment to maintain the patient's quality of life, as well as to consider conservative management.^[3,14,19,20]^ Treatment is focused on relieving pain, improving movement restriction, and preserving function. As each tumor type will behave uniquely, options include analgesia, radiotherapy, amputation, wide excision, curettage, cementation, radiotherapy, and systemic chemotherapy.^[1,2,6,18,19,21]^

The true incidence of this entity may be higher that what is reported because of subclinical lesions, unreported cases, undiagnosed tumors in severely debilitated patients, and lack of biopsy to confirm the metastasis.^[1,2]^ Therefore, in patients presenting with non-mechanical hand or thumb pain and swelling, as well as a mass with a history of prior malignancy, a malignant process should be considered.^[3]^

## 4 Conclusion

Acrometastases are infrequent presentations of widespread disease; most of the time, the evolution is aggressive, and the prognosis is poor with limited survival. Metastases in unusual sites are a diagnostic challenge, and there is no standardized treatment. Diagnostic and timely treatment can improve the prognosis of these patients and might preserve their quality of life. The best way to find causes, improve the diagnosis, and seek the best treatment options is the publication and diffusion of this kind of case.

## Author contributions

**Clinical team:** David Cantu-de Leon, Alma Astorga Ramos

**Conceptualization:** David Cantu-de Leon, Lenny Gallardo-Alvarado, Alma Astorga Ramos

**Data curation:** Lenny Gallardo-Alvarado, Alma Astorga Ramos, Delia Perez-Montiel, David Francisco Cantu-De Leon.

**Formal analysis:** Delia Perez-Montiel, Rebeca Ramirez-Morales.

**Investigation:** Lenny Gallardo-Alvarado, Rebeca Ramirez-Morales, Erick Diaz, David Francisco Cantu-De Leon, Delia Perez-Montiel.

**Literature review:** Lenny Gallardo-Alvarado, Rebeca Ramirez-Morales, Erick Diaz

**Methodology:** Lenny Gallardo-Alvarado, Alma Astorga Ramos, Delia Perez-Montiel, Rebeca Ramirez-Morales, Erick Diaz.

**Pathologic diagnosis:** Delia Perez-Montiel

**Project administration:** David Francisco Cantu-De Leon.

**Resources:** Lenny Gallardo-Alvarado, Alma Astorga Ramos, Delia Perez-Montiel, Rebeca Ramirez-Morales, Erick Diaz, David Francisco Cantu-De Leon.

**Supervision:** Lenny Gallardo-Alvarado, David Francisco Cantu-De Leon. David Francisco Cantu-De Leon

**Validation:** Lenny Gallardo-Alvarado, Alma Astorga Ramos, Delia Perez-Montiel, David Francisco Cantu-De Leon, Rebeca Ramirez-Morales.

**Writing – original draft:** Lenny Gallardo-Alvarado, Erick Diaz, David Cantu-De Leon.

**Writing – review & editing:** Lenny Gallardo-Alvarado, Erick Diaz, David Cantu-de Leon.

## References

[1] Hayden RJ Sullivan LG Jebson PJL The hand in metastatic disease and acral manifestations of paraneoplastic syndromes. Hand Clin 2004;20:335–43. 15275692 10.1016/j.hcl.2004.03.010

[2] Afshar A Farhadnia P Khalkhali H Metastases to the hand and wrist: An analysis of 221 cases. J Hand Surg Am 2014;39:923–32. 24612837 10.1016/j.jhsa.2014.01.016

[3] Morris G Evans S Stevenson J Bone metastases of the hand. Ann R Coll Surg Engl 2017;99:563–7. 28853594 10.1308/rcsann.2017.0096 PMC5697042

[4] Falk DP Scully R Moss D Pathologic tuft fracture in a thumb: a rare presentation of metastatic endometrioid ovarian carcinoma: a case report and review of the literature. JBJS Case Connect 2017;7:e50. 29252880 10.2106/JBJS.CC.16.00261

[5] Kerin R The hand in metastatic disease. J Hand Surg Am 1987;12:77–83. 3543107 10.1016/s0363-5023(87)80164-8

[6] Ward R Metastatic bone disease: Forearm, hand. Metastatic Bone Disease: An Integrated Approach to Patient Care.. New York, NY: Springer New York; 2015.

[7] Mavrogenis AF Mimidis G Kokkalis ZT Acrometastases. Eur J Orthop Surg Traumatol 2014;24:279–83. 24013815 10.1007/s00590-013-1311-1

[8] Fan G Xie Y Pei X Renal metastasis from cervical carcinoma presenting as a renal cyst: a case report. Oncol Lett 2015;10:2761–4. 26722238 10.3892/ol.2015.3690 PMC4665838

[9] Pfaendler KS Tewari KS Changing paradigms in the systemic treatment of advanced cervical cancer. Am J Obstet Gynecol 2016;214:22–30. 26212178 10.1016/j.ajog.2015.07.022 PMC5613936

[10] Panek G Gawrychowski K Sobiczewski P Results of chemotherapy for pulmonary metastases of carcinoma of the cervix in patients after primary surgical and radiotherapeutic management. Int J Gynecol Cancer 2007;17:1056–61. 17466044 10.1111/j.1525-1438.2007.00879.x

[11] Thanapprapasr D Nartthanarung A Likittanasombut P Bone metastasis in cervical cancer patients over a 10-year period. Int J Gynecol Cancer 2010;20:373–8. 20375800 10.1111/IGC.0b013e3181d4a0a1

[12] Pasricha R Tiwari A Aggarwal T Carcinoma of uterine cervix with isolated metastasis to fibula and its unusual behavior: report of a case and review of literature. J Cancer Res Ther 2006;2:79–81. 17998682 10.4103/0973-1482.25857

[13] Yoon A Choi CH Kim H-J Contributing factors for bone metastasis in uterine cervical cancer. Int J Gynecol Cancer 2013;23:1311–7. 23839246 10.1097/IGC.0b013e31829da127

[14] Chen C-H Chao K-C Wang P-H Advanced cervical squamous cell carcinoma with skin metastasis. Taiwan J Obstet Gynecol 2007;46:264–6. 17962107 10.1016/S1028-4559(08)60031-5

[15] Behtash N Ghaemmaghami F Yarandi F Cutaneous metastasis from carcinoma of the cervix at the drain site. Gynecol Oncol 2002;85:209–11. 11925148 10.1006/gyno.2001.6559

[16] Benoulaid M Elkacemi H Bourhafour I Skin metastases of cervical cancer: two case reports and review of the literature. J Med Case Rep 2016;10:265. 27663996 10.1186/s13256-016-1042-0 PMC5035488

[17] Skenderi F Chikha A Ibisevic N Skeletal muscle metastases from squamous cell carcinoma of the cervix. Int J Gynecol Pathol 2017;36:95–100. 27391272 10.1097/PGP.0000000000000298

[18] Kumar N Kumar R Bera A Palliative and supportive care in acrometastasis to the hand: case series. Indian J Palliat Care 2011;17:241–4. 22346051 10.4103/0973-1075.92347 PMC3276824

[19] Heloisa de Andrade Carvalho PWCT and TYT. Thumb metastasis from small cell lung cancer treated with radiation. Rev Hosp Clín Fac Med S Paulo 2002;57:283–6. 12612761 10.1590/s0041-87812002000600007

[20] Muñoz-Mahamud E Combalia A Carreño A Five cases of acrometastasis to the hand from a carcinoma and review of the literature. Hand Surg Rehabil 2017;36:12–6. 28137435 10.1016/j.hansur.2016.10.211

[21] Machado V San-Julian M Pronóstico y tratamiento de las acrometástasis: estudio observacional de 35 casos tratados en un único centro. Rev Esp Cir Ortop Traumatol 2019;63:49–55. 30612612 10.1016/j.recot.2018.05.001

